# Feeding practices and micronutrient status of children aged 0–36 months in Thulamela Municipality, Limpopo province

**DOI:** 10.4102/hsag.v27i0.1973

**Published:** 2022-10-24

**Authors:** Anzani Mugware, Selekane A. Motadi, Lindelani F. Mushaphi

**Affiliations:** 1 Department of Nutrition, Faculty of Health Sciences, University of Venda, Thohoyandou, South Africa

**Keywords:** breastfeeding, complementary feeding, vitamin A, iron deficiency, micronutrients

## Abstract

**Background:**

Micronutrient deficiency continues to be a major public health problem affecting infants and young children under 5 years of age worldwide.

**Aim:**

The study aims to investigate feeding practices and micronutrient status of children aged 0–36 months.

**Setting:**

The study was conducted at government clinics located in Thulamela Municipality, which is one of the local municipalities in Vhembe District, Limpopo province.

**Methods:**

A cross-sectional survey was conducted. A total of 250 mothers with children aged 0–36 months were enrolled. A structured questionnaire was used to interview the mothers. Biochemical measurements of children were assessed using standard procedures.

**Results:**

Only 7.6% of children were exclusively breastfed for 6 months. Majority (87.5%) of the children were introduced to complementary foods before 6 months and 98.8% of the children had a low dietary diversity score of less than four, while 9.2% had a dietary diversity score of more than four. The prevalence of vitamin A deficiency, anaemia and iron deficiency was 21.7%, 53.6% and 13.1%, respectively. For mothers who initiated breastfeeding immediately after delivery, the odds of children having low ferritin were 0.11 times, as compared to children who were initiated breastfeeding a day after delivery (odds ratio = 0.11; 95% confidence interval = 0.015–0.812).

**Conclusion:**

Most of the children were introduced to complementary foods earlier than 6 months of age. Infant feeding practices were associated with micronutrients status.

**Contribution:**

The study contributes to the body of literature on feeding practices and the micronutrient status of children.

## Background

Micronutrient deficiency continues to be a major public health problem affecting infants and young children under 5 years of age worldwide (Bailey, West & Black 2015). Vitamin A and iron are the most common micronutrient deficiencies that affect more than 2 billion people worldwide, with 190 million children being vitamin A deficient and 600 million iron deficient (United Nation System Standing committee on Nutrition 2017). About 38.7% of children in South Africa were reported to be anaemic, while 63.6% of children under 5 years were reported to be vitamin A deficient (National Food Consumption Survey-Fortification Baseline [NFCS-FB-I] 2005). In 2012, the South Africa National Health and Nutrition Examination Survey (SANHANES-1) found that 43.6% of children were suffering from vitamin A deficiency, while 11% were suffering from iron deficiency (SANHANES-1 2012). In Limpopo province, Mushaphi et al. (2015) reported that 7.7% of children under the age of 5 years were vitamin A deficient, while 3.5% were iron deficient.

The World Health Organization (WHO 2021) recommends early initiation of breastfeeding within an hour after delivery, exclusively breastfeeding for the first 6 months of life, followed by the introduction of complementary foods at 6 months while continuing breastfeeding at least 2 years or even beyond. In South Africa, the rate of exclusive breastfeeding for the first 6 months remains below 10%, while introduction of solid and semisolid foods before 6 months is popular practice (SANHANES-1 2012). Micronutrient malnutrition in the early stage of life has been directly associated with poor infant feeding practices and high rates of infectious diseases, especially in developing countries such as South Africa (Bailey et al. 2015; Berti, Faber & Smuts 2014; Mushaphi et al. 2017). Micronutrient malnutrition during childhood leads to growth retardation, delaying of mental development and impairment of intellectual level (Ma’alin et al. 2016).

The South African government implemented several initiatives such as vitamin A supplementation, deworming and food fortification to prevent micronutrient deficiencies. However, micronutrient deficiency remains a major public health problem affecting children under 5 years of age in South Africa (SANHANES-1 2012). According to the South African Vitamin A Consultative Group (Labadarios et al. 1995), the National Department of Health (2007) and the National Food Consumption Survey (2005), vitamin A and iron deficiency have doubled between 1994 and 2005 in South Africa. The high prevalence of vitamin A and iron among children under 5 years was reported in Limpopo province (NFCS-FB-I 2005; SANHANES-1 2012). Inappropriate feeding practices such as the early introduction of complementary foods and mixed feeding were major contributors to child malnutrition in Limpopo province (Mamabolo et al. 2004; Mushaphi et al. 2008). Therefore, it is important to investigate the feeding practices and micronutrient nutritional status of children aged 0–36 months in Thulamela Municipality of Vhembe District.

## Methods and materials

### Study design

This cross-sectional study assessed the feeding practices and micronutrient status of children. In this research, data were collected at one point. The quantitative method was used to gather information. A quantitative method is a technique used to gather numerical data and anything that is measurable.

### Study setting

The study was conducted in Thulamela Municipality, which is one of the four local municipalities found in the Vhembe District Municipality of the Limpopo province. In 2016, Thulamela had an estimation of 497 237 population, with 163 884 being children from 0 to 4 years (Statistics SA 2016). According to the District Health Information System (2009), Thulamela Municipality has approximately 2234 children who are attending well-baby clinic services. Health care services in Thulamela Municipality are delivered by three hospitals, one specialised psychiatric hospital, 43 clinics and six mobile clinics. Thulamela Municipality comprises six local areas: Ha-Madala, Tshaulu, Sibasa, Mutale, Shayandima and William Eadie.

### Study population and sampling

This study enrolled mothers with children aged 0–36 months in Thulamela Municipality. The mothers in this study were the main sources of information. The age category of 0–36 months of children was chosen in line with WHO guidelines for infant and young child feeding practices (IYCF). All six local areas in Thulamela municipality were regarded as a cluster in the study. Slovin’s formula: *n* = N/(1 + (N × e^2^)) was used to calculate the number of clinics (18) and the total number of participants (250). Simple random sampling was used to select three clinics per cluster to make 18 clinics. Convenience sampling technique was used to select 250 children who were on breastfeeding and complementary feeding during a well-baby clinic visit. All mothers with children aged 0–36 months who agreed to participate and signed the consent form were included in the study. In a case where the mother was younger than 18 years, guardians were requested to sign the informed consent form. The mother of the child was also given an assent form if she agreed that the child should form part of the study. Children who were severely ill and required emergency attention were excluded from the study. Children whose parents or guardians did not give consent were excluded from the study.

### Instrument development

The structured questionnaire was used to collect data. The questionnaire was developed based on the aim and objectives of the study. The sociodemographic questions were adapted from the questionnaire of a study titled ‘Infant feeding practices and anthropometric status of children aged 3–24 months at Mukula village, Vhembe District, Limpopo Province of South Africa’ (Mugware 2014). The questionnaire consisted of the following sections: sociodemographic characteristics (Section A), breastfeeding practices (Section B), complementary feeding practices (Section C) and nutritional status (Section D). The breastfeeding and complementary feeding practices questionnaire was developed based on the WHO’s indicators for assessing IYCF (WHO 2008, 2010) and the guiding principles for complementary feeding (WHO 2003). The questionnaire was developed in English and translated into the local language (Tshivenda) by an expert from the MER Mathivha Language Centre at the University of Venda and back to English to ensure accuracy of the translation. The questionnaire was given to the supervisors to check for content and appropriateness. In addition, the questionnaire was given to nutritionists and other professionals in the Faculty of Health Sciences to determine the appropriateness and completeness of the questionnaire.

### Validity and reliability

The questionnaire was piloted before data collection to ensure validity. Mothers were interviewed using the local language (Tshivenda) to ensure that accurate information is obtained. All the questionnaires were checked at the end of each day to ensure accuracy, quality and completeness. To check for reliability, 10% of the questionnaires were randomly selected and 47 mothers were interviewed again one week after the initial round of data collection. This quality control interview was conducted on a different day from the initial interview by a different interviewer. To ensure quality control, data were analysed in the same way as the original data for comparison.

### Data collection procedure

The questionnaire was used to interview mothers on feeding practices. To assess minimum dietary diversity (MDD) of children, mothers were interviewed using 24-h recall. Mothers were asked to name all the foods that the child had consumed during the previous 24 h. The researcher used food cards, pictures and models to assist mothers to remember the food items they fed their children in the previous 24 h (FAO 2016). The information gathered on the dietary diversity score assessed the type of food groups consumed by the child. The score ranged from four to seven types of food groups. A score of less than four out of seven indicated a low dietary diversity, while a score of more than four indicated adequate dietary diversity (UNICEF 2007).

A phlebotomist from Ampath Laboratory collected 138 blood samples from 18 PHC facilities in Thulamela Municipality. Blood samples were drawn from children following the standard procedures stipulated in the WHO guidelines for drawing blood (WHO 2010). The blood samples were collected during the data collection. The blood samples were analysed using standard procedures in the Ampath Pathology Laboratory (Drs Du Buisson, Kramer Inc./Ing.). The blood samples were used to assess the vitamin A and iron status of children. The following cut-off points were used to assess micronutrient deficiency among children; serum retinol concentration < 10 µg/dL (NFCS-FB-I 2005), anaemia Hb *<* 11.0 g/dL (WHO 2011a), serum ferritin < 12 µg/L (WHO 2011b), serum iron < 60 µg/dL, serum transferrin < 2.0 g/L and saturation transferrin < 15% (Gibson 2005).

### Data analysis

The data obtained were cleaned, coded and entered on a Microsoft (MS) Excel spreadsheet, then exported to IBM Statistical Package for Social Sciences version 26 for analysis (SPSS Inc., Chicago, Illinois). Descriptive statistics were performed for all variables in the study. The categorical variables were presented as frequencies (*n*) with percentage (%) of the total study sample. Continuous variables were presented as mean ± standard deviation (SD) for normally distributed data. A chi-square test for the categorical variables was performed. The chi-square test was used to discover the relationship between two categorical variables. Logistic regression analysis was performed to explore associations between micronutrients and feeding practices. The crude odds ratio (COR) and adjusted odds ratios (AOR) together with their corresponding 95% confidence intervals were computed and interpreted accordingly. A *p* < 0.05 was considered to declare a result statistically significant.

### Ethical considerations

Ethical clearance was obtained from the University of Venda Research Ethics Committee (reference number: SHS/19/NUT/01/1503) and the Provincial Department approved the study of the Health Research Committee. The study was performed in accordance with the principles of the Declaration of Helsinki, good clinical practices and the laws of South Africa. Parents of children were informed about the minimal risk of needle-prick injury: the taking of blood can be uncomfortable and may leave a burning sensation for a short time. Qualified phlebotomists performed the task to minimise the risks. Only two attempts were made to draw blood from the child’s body. If, on the second attempt, the phlebotomists could not succeed in drawing the blood from the child, the process was stopped. An oral and written explanation of the study, including possible risks, was provided to the parents before the commencement of the study. Mothers were given consent forms to sign for their children to participate in the study. The consent form included the respondent’s right to withdraw from the study. The participants were assured of confidentiality and anonymity of information. Codes were used instead of the names of participants.

## Results

### Sociodemographic characteristics of the study participants

A total number of 250 mothers with children aged 0–36 months were enrolled in this study. The mean (± SD) age of children in months 10.53 (8.39). More than half (52.2%) of the children were male, while 47.6% were female. The mean (± SD) age of mothers in years was 27.79 (± 7.13 SD). More than half (54%) of the mothers were single, while 37.6% were married. More than one-third (37.2%) of the mothers had passed their Grade 12, while 16.8% had tertiary education. Three-quarters of mothers (75.2%) were unemployed (Table 1).

**TABLE 1:** Sociodemographic characteristics of the study participants (*n* = 250).

Characteristics	Frequency (*n*)	Percentage (%)
**Age of child (months)**		
0–6	101	40.4
7–12	72	28.8
13–24	55	22.0
25–36	22	8.8
**Gender**		
Male	131	52.4
Female	119	47.6
**Maternal age (years)**		
≤ 19	31	12.4
20–29	123	49.2
30–39	88	35.2
≥ 40	8	3.2
**Marital status**		
Single	135	54.0
Married	94	37.6
Divorced	5	2.0
Living with partner	9	3.6
Separated	7	2.8
**Education level**		
Never attended	1	0.4
Grade 1–4	2	0.8
Grade 5–7	6	2.4
Grade 8–10	48	19.2
Grade 11–12	58	23.2
Passed grade 12	93	37.2
Tertiary	42	16.8
**Employment status**		
Employed	62	24.8
Unemployed	188	75.2

#### Breastfeeding practices

A majority (96%) of mothers initiated breastfeeding within 1 h after birth. Almost all (95.2%) mothers were assisted by nurses to initiate breastfeeding. About 7.6% of mothers exclusively breastfed their children up to 6 months. Almost three-quarters of mothers were still breastfeeding at the time of the interview, while 26% were no longer breastfeeding. Of all the mothers, 68.8% exclusively breastfed their children for 2 months, while 23.6% breastfed their children for 3–5 months. About 38.8% of mothers chose to breastfeed because it is the perfect food for a child. About 16.3% of mothers stopped breastfeeding before 12 months, and the main reason for stopping breastfeeding was going back to school or work. More than one-third (36.4%) of children received infant formula (Table 2).

**TABLE 2:** Breastfeeding practices (*n* = 250).

Variables	Frequency (*n*)	Percentages (%)
**Time of initiation breastfeeding**		
Within 1 h after birth	240	96.0
A day after birth	8	3.2
Week after birth	2	0.8
**Assistance of initiation of breastfeeding**		
Nurse	237	95.2
Medical doctor	13	5.6
**Sources of breastfeeding information**		
Healthcare professionals	209	91
Parents or parents-in-law	2	0.9
Media (magazine, radio and television)	3	1.3
Mom-connect	15	6.6
**Duration of exclusive breastfeeding**		
0–2 months	172	68.8
3–5 months	59	23.6
6 months	19	7.6
**Breastfeeding status**		
Still breastfeeding	185	74
Stopped breastfeeding	65	26
**Reasons for choosing breastfeeding**		
Perfect food for a child	97	38.8
Protect child against diseases	75	30.0
Free	3	1.2
Bonding between mother and child	10	4.0
**Breastfeeding frequency**		
On-demand	112	44.8
On schedule time	23	9.2
At your own time	50	20
**Age of stopping breastfeeding**		
Less than 1 month	7	2.8
2–5 months	17	6.8
6–12 months	18	7.2
13–24 months	23	9.2
**Reasons for stopping breastfeeding**		
Medical condition	13	5.2
Child refused breast	10	4.0
Going back to school or work	27	10.8
Not enough breastmilk	8	3.2
Another pregnancy	3	1.2
Was the right time to stop breastfeeding	4	1.6
**Child receive infant formula**		
Yes	91	36.4
No	159	63.6

*Source*: WHO (2003; 2008; 2010)

Note: Please see the full reference list of the article, Mugware, A., Motadi, S.A. & Mushaphi, L.F., 2022, ‘Feeding practices and micronutrient status of children aged 0–36 months in Thulamela Municipality, Limpopo Province’, Health SA *Gesondheid* 27(0), a1973. https://doi.org/10.4102/hsag.v27i0.1973, for more information.

### Complementary feeding practices

Nearly three-quarters (73.6%) of children had been introduced to complementary foods, while 26.4% had not been introduced to complementary foods at the time of the interview. A majority (87.5%) of children were introduced to complementary food before 6 months of age, such as soft porridge. Furthermore, 91.2% of children were given water before 6 months of age. The main reason for giving water was that the child cried a great deal (70.1%), advised by parents (11.4%). About 28% of children consumed vitamin A–rich food in the past 24 h, while 22.6% consumed iron-rich foods. The MDD was attained by 9.2% of children (Table 3).

**TABLE 3:** Complementary feeding (*n* = 184).

Variables	Frequency (*n*)	Percentages (%)
**Age of introducing complementary foods**		
1 month	7	3.8
2–3 months	35	19.0
4–5 months	119	64.7
6 months	23	12.5
**Age of introducing water**		
0–2 months	43	17.2
3–5 months	142	74
6 months and above	21	8.4
**Reasons for introducing complementary foods**		
Child cries a great deal	129	70.1
Insufficient breastmilk	15	8.2
Advised by parents	21	11.4
Advised by health professionals	17	9.2
It was the right time	2	1.1
**Sources of complementary feeding information**		
Health worker (nurse, dietician or nutritionist)	94	87.9
Parents or parents-in-law	7	6.5
Media (newspaper, radio and television)	5	4.7
Peers (friends, colleagues).	1	0.9
**Dietary diversity score**		
< 4 food groups	167	90.8
≥ 4 food groups	17	9.2
**Consumption of vitamin A–rich foods**		
Consumed	52	28.0
Not consumed	134	72.0
**Consumption iron-rich foods**		
Consumed	42	22.6
Not consumed	144	77.4

*Source*: WHO (2008; 2010a) and FAO & FHI 360, (2016:82)

Note: Please see the full reference list of the article, Mugware, A., Motadi, S.A. & Mushaphi, L.F., 2022, ‘Feeding practices and micronutrient status of children aged 0–36 months in Thulamela Municipality, Limpopo Province’, *Health SA Gesondheid* 27(0), a1973. https://doi.org/10.4102/hsag.v27i0.1973, for more information.

### Micronutrient status

The mean (± SD) serum retinol concentration was 25.17 µg/dL (± 6.289). The prevalence of vitamin A among children was 21.7% (< 10 µg/dL). The mean (± SD) haemoglobin concentration was 10.98 g/dL (± 1.145 SD). The prevalence of anaemia was 53.6%. The mean (± SD) serum iron was 47.33 µg/dL (± 21.97 SD). Close to a quarter (24%) of children had iron depletion. Serum ferritin’s mean (± SD) serum was 33.88 µg/L (± 29.33 SD). The prevalence of iron deficiency was 13% among children. The mean (± SD) serum transferrin was 2.98 g/L (± 29.33 SD). About 2.9% of children had mild depletion. The mean (± SD) saturation transferrin was 51.48% (± 43.22 SD). More than one-third (37%) of children had low saturation transferrin (Table 4).

**TABLE 4:** Micronutrient status of children.

Micronutrient indicators	Cut-off values	*n* = 138	(%)	Mean ± SD
**Serum retinol concentration**				
Vitamin A deficiency	< 10 μg/dL	2	1.4	-
Marginal vitamin A status	10 μg/dL – 19.9 μg/dL	28	20.3	25.17 ± 6.289
Adequate status	20 μg/dL – 29.9 μg/dL	75	54.3	-
Normal or well-nourished status	> 30 μg/dL	33	23.9	-
**Haemoglobin concentrations**				
Severe anaemia	< 7.0 g/dL	0	0	-
Moderate anaemia	7.0 g/dL – 9.9 g/dL	29	21	10.98 ± 1.145
Mild anaemia	10.0 g/dL – 10.9 g/dL	45	32.6	-
Normal	≥ 11 g/dL	64	46.4	-
**Serum iron**				
Depletion	< 60 μg/dL	24	17.4	47.33 ± 21.97
Normal	≥ 115 μg/dL	114	82.6	-
**Serum ferritin**				
Iron deficiency	< 12 μg/L	18	13.1	33.88 ± 29.33
Normal iron stores	≥ 12 μg/L	120	86.9	-
**Serum transferrin**				
Severe depletion	< 1.0 g/L	0	0	-
Mild depletion	1.5 g/L – 2.0 g/L	4	2.9	2.98 ± 29.33
Normal	> 2.0 g/L	134	97.1	-
**Saturation transferrin**				
Low	< 15%	51	37.0	51.48 ± 43.22
Normal	10% – 20%	87	63.0	-

*Source*: NFCS-FB-I (2005) and WHO (2011a; 2011b)

Note: Please see the full reference list of the article, Mugware, A., Motadi, S.A. & Mushaphi, L.F., 2022, ‘Feeding practices and micronutrient status of children aged 0–36 months in Thulamela Municipality, Limpopo Province’, *Health SA Gesondheid* 27(0), a1973. https://doi.org/10.4102/hsag.v27i0.1973, for more information.

SD, standard deviation.

### Association between micronutrient status and infant feeding practices of children

Table 5 shows the association between micronutrient and infant feeding practices. Children who were still breastfeeding were more likely to have normal haemoglobin (73.5%) as compared to those who were no longer breastfeeding (61.5%) (*p* = 0.045). Most children (93.4%) who were on infant formula milk were more likely to have normal vitamin A status as compared to children who were not given infant formula milk (84.9%) (*p* = 0.047). One-third (33%) of children who were given water were more likely to have anaemia as compared to those who were not given water (13.6%) (*p* = 0.011). Children who were given water before 6 months were more likely to have anaemia as compared to those who were not given water (*p* = 0.001). There was a significant relationship between children who received water before 6 months and iron deficiency (serum ferritin) (*p* = 0.04). Children who were introduced to complementary foods were more likely to have anaemia than those who were not introduced to complentary foods (*p* = 0.001). There was a significant relationship between children who were not introduced to complementary foods and low saturation (*p* = 0.021).

**TABLE 5:** The association between infant feeding practices and micronutrient status of children.

Variables	Haemoglobin				Vitamin A				Haemoglobin				Haemoglobin				Serum ferritin				Haemoglobin				Saturation				df	*p*
	Anaemia		Normal		VAD		Normal		Anaemia		Normal		Anaemia		Normal		ID		Normal		Anaemia		Normal		Low		Normal			
	*n*	%	*n*	%	*n*	%	*n*	%	*n*	%	*n*	%	*n*	%	*n*	%	*n*	%	*n*	%	*n*	%	*n*	%	*n*	%	*n*	%		
**Still breastfeeding**																														
Yes	49	26.5	136	73.5	-	-	-	-	-	-	-	-	-	-	-	-	-	-	-	-	-	-	-	-	-	-	-	-	1	0.045
No	25	38.5	40	61.5	-	-	-	-	-	-	-	-	-	-	-	-	-	-	-	-	-	-	-	-	-	-	-	-		
**Child received infant formula milk**																														
Yes	-	-	-	-	6	6.6	85	93.4	-	-	-	-	-	-	-	-	-	-	-	-	-	-	-	-	-	-	-	-	1	0.047
No	-	-	-	-	24	15.1	135	84.9	-	-	-	-	-	-	-	-	-	-	-	-	-	-	-	-	-	-	-	-		
**Introduction of water**																														
Yes	-	-	-	-	-	-	-	-	68	33.0	138	67.0	-	-	-	-	-	-	-	-	-	-	-	-	-	-	-	-	1	0.011
No	-	-	-	-	-	-	-	-	38	86.4	6	13.6	-	-	-	-	-	-	-	-	-	-	-	-	-	-	-	-		
**Age of introducing water**																														
6 months and below	-	-	-	-	-	-	-	-	-	-	-	-	65	35.1	120	64.9	-	-	-	-	-	-	-	-	-	-	-	-	1	0.001
Above 6 months	-	-	-	-	-	-	-	-	-	-	-	-	9	13.8	56	86.2	-	-	-	-	-	-	-	-	-	-	-	-		
**Age of introducing water**																														
6 months and below	-	-	-	-	-	-	-	-	-	-	-	-	-	-	-	-	17	9.2	168	90.8	-	-	-	-	-	-	-	-		
Above 6 months	-	-	-	-	-	-	-	-	-	-	-	-	-	-	-	-	1	1.5	64	98.5	-	-	-	-	-	-	-	-	1	0.040
**Introduction of CF**																														
Yes	-	-	-	-	-	-	-	-	-	-	-	-	-	-	-	-	-	-	-	-	65	35.3	119	64.7	-	-	-	-	1	0.001
No	-	-	-	-	-	-	-	-	-	-	-	-	-	-	-	-	-	-	-	-	9	13.6	57	86.4	-	-	-	-		
**Introduction of CF**																														
Yes	-	-	-	-	-	-	-	-	-	-	-	-	-	-	-	-	-	-	-	-	-	-	-	-	44	23.9	140	76.1	1	0.021
No	-	-	-	-	-	-	-	-	-	-	-	-	-	-	-	-	-	-	-	-	-	-	-	-	7	10.6	59	89.4		

*Source:* Gibson (2005), NFCS-FB-I (2005) and WHO (2008; 2010; 2011a; 2011b)

Note: Please see the full reference list of the article, Mugware, A., Motadi, S.A. & Mushaphi, L.F., 2022, ‘Feeding practices and micronutrient status of children aged 0–36 months in Thulamela Municipality, Limpopo Province’, *Health SA Gesondheid* 27(0), a1973. https://doi.org/10.4102/hsag.v27i0.1973, for more information.)

CF, complementary foods; VAD, vitamin A deficiency; ID, iron deficiency.

### Association between micronutrient and feeding practices

Table 6 shows results from logistic regression of associations between feeding practices and micronutrient status of children. For children who were initiated on breastfeeding immediately after delivery, the odds of having iron deficiency were 0.11 times as compared to those who were initiated breastfeeding a day after delivery (OR = 0.111; 95% CI = 0.015–0.812). Since the OR is less than 1, it means children who were initiated on breastfeeding immediately after delivery have a low chance of having iron deficiency, as compared to children who were initiated on breastfeeding a day after delivery. For children who were given infant formula milk, the odds of having iron deficiency were 0.22 times, as compared to those who were not given infant formula milk (OR = 0.216; 95% CI = 0.046–1.018). For children who were given water before the age of 6 months, the odds of having anaemia were about 3 times, compared to those given water after 6 months (OR = 3.081; 95% CI = 1.005–9.441).

**TABLE 6:** Association between infant feeding practices and micronutrient status of children.

Variables	Serum ferritin			Haemoglobin		
	OR	95% CI	*p*	OR	95% CI	*p*
**Breastfeeding initiation**						
Immediately after delivery	0.111	0.015–0.812	0.030	-	-	-
**Is your baby on infant formula milk?**						
Yes	0.216	0.046–1.018	0.053	-	-	-
**Age of introducing water**						
6 months and below	-	-	-	3.081	1.005–9.441	0.049

OR, odds ratio; CI, confidence interval.

Significance at *p* < 0.05.

## Discussion

The first 1000 days of life from conception to 2 years of age are a critical period to promote growth and development and a period in which malnutrition can occur because of poor feeding practices that may have long-lasting effects in later life (Schwarzenberg et al. 2018). The UNICEF (2007) recommended that breastfeeding should be initiated within the first hour after birth. The findings in this study revealed that a majority (96%) of mothers initiated breastfeeding within an hour after birth, which is in line with UNICEF (2007) recommendations. Similar findings were reported in previous studies conducted in southern Ethiopia (Beyene et al. 2016), eastern Sudan (Hassan et al. 2018), Bangladesh (Haider & Saha 2016) and Malawi (Chipojola et al. 2020), where the rates of initiating breastfeeding within an hour after birth were 83.7%, 87.2%, 94% and 95.4%, respectively. However, Ghana (39%) and the United States of America (39%) reported low rates of breastfeeding initiation as compared to the current study (Fosu-Brefo & Arthur 2015; Thomson et al. 2017). According to Olufunlayo et al. (2019), factors such as maternal education and maternity services determine the rate of early initiation of breastfeeding within an hour, especially in low- and middle-income countries. More than 90% of mothers in the current study and previous studies (Chipojola et al. 2020; Haider & Saha 2016) initiated breastfeeding within the recommended time by WHO, which indicates very good practices.

The UNICEF (2007) recommended that infants should be exclusively breastfed for the first 6 months after birth. Victora et al. (2016) indicated that exclusive breastfeeding for the first 6 months is the most effective preventive intervention for reducing child morbidity and mortality. Despite evidence of many health benefits of exclusive breastfeeding for the first 6 months, the results of this study showed that only 7.6% of children were exclusively breastfed for the first 6 months. The rate of exclusive breastfeeding in this study is lower than the national rate of exclusive breastfeeding of 32% in 2016, as shown in the South African Demographic and Health Survey (SADHS 2016). In a study conducted in the same district, Mushaphi et al. (2017) indicated that none of the children were exclusively breastfed for 6 months. Several studies revealed that mixed feeding and early introduction of solid foods are common practices in South Africa (Budree et al. 2017; Doherty et al. 2012; Mushaphi et al. 2017). Lack of maternal self-efficacy, returning to work or school, poor experiences of breastfeeding and perceptions of insufficient milk are the most common barriers to exclusive breastfeeding for the first 6 months (Doherty et al. 2012; Du Plessis 2013; Rollins et al. 2016).

In the current study, most mothers indicated that returning to school or work, influence by the family members and insufficient milk were the main reasons for introducing complementary foods or fluids. A similar observation was made in a study carried out in KwaZulu-Natal by Horwood et al. (2018), where several factors such as going back to work or school and HIV status were associated with early breastfeeding cessation. Poor breastfeeding rate could be because when infants are given water and complementary foods at an early stage, they are likely to stop breastfeeding because of nipple confusion if given an artificial teat, or the child is full of other foods or fluid and ends up less interested in the breast (Kavitha, Nadhiya & Parimalavalli 2014). Furthermore, at this stage, the infant’s gastrointestinal and neurodevelopmental systems and the kidneys are still too immature to handle solid food and water. Provision of breastfeeding support from health workers, community health workers, peer supporters and others in the family or community have consistently been associated with improved feeding practices (Rollins et al. 2016; Shifraw, Worku & Berhane 2015; Tylleskar et al. 2011).

Despite Vhembe district being rich in fruits and vegetables that are excellent sources of vitamin A, the prevalence of vitamin A deficiency in the current study was 21.7%. The prevalence of vitamin A in the current study is lower than all national surveys conducted in South Africa (Labadarios et al. 1995, 2007; SANHANES-1 2012). High prevalence of vitamin A in the current study may be attributed to children being fed maize meal soft porridge without introducing micronutrient-rich food. Consumption of a monotonous diet based on starchy food with limited vitamin and mineral-rich food consumption is likely to increase the risk of micronutrient deficiency in children (Kavitha et al. 2014; Mitchodigni et al. 2018; Ntila et al. 2017). Low dietary intake of vitamin A–rich food, rapid growth and repeated infections are the major causes of vitamin A deficiency, especially in early childhood (Gibson 2005). The timing of introducing complementary feeding is nutritionally crucial, as requirements for nutrients are greater during the second half of infancy (Papoutsou et al. 2018). South Africa’s vitamin A supplementation coverage remains very low. Mpumalanga province had the lowest vitamin A supplementation coverage (30%), followed by Limpopo province (32%), Northern Cape province (32%) and North West province 33%. However, the current study did not determine vitamin A supplementation coverage (District Health Information System 2009).

More than (53.6%) of children in the current study were anaemic. This may be because no iron-rich foods were introduced during complementary feeding, and children had low dietary diversity scores in the current study. Congruently, a study conducted in Limpopo province found 75% of children were anaemic (Heckman et al. 2010). Even though South Africa had legislated fortification of flour and maize to reduce micronutrient malnutrition, food introduced during childhood includes small amounts of iron, rich with a high content of substances such as phytates and oxalates, tannins and fibre that interfere with iron absorption (Mitchodigni et al. 2018). Lack of knowledge about the importance of food groups, food insecurity, poor food choices and limited food availability may limit the inclusion of food rich in micronutrients during the introduction of complementary food (Mitchodigni et al. 2018). In low-income rural villages of Africa, many people have limited access to diets that meet their daily macro- and micronutrient requirements, resulting in malnutrition, particularly underweight. Many low-income households in rural areas of South Africa are highly dependent on social grants for survival (old-age pension and child support grants), and this makes it difficult for them to purchase food that could meet their daily requirements of nutrients because of the high cost of foods, especially protein-rich food (Labadarios et al. 1995; SADHS 2016; SANHANES-1 2012). Insufficient consumption of iron leads to poor physical work capacity, growth retardation and alterations in the neurological function being compromised (Brown et al. 2004; SANHANES-1 2012). Most studies have found associations between iron-deficiency anaemia and poor cognitive and motor development and behavioural problems (Bailey et al. 2015; Batool et al. 2022).

Iron deficiency, as indicated by low serum ferritin (12 µg/dL) and serum transferrin (< 2.0 g/L), was present in 13% and 2.9% of children, respectively. The prevalence of iron deficiency (serum ferritin < 12 µg/dL) was higher when compared to the NFCS-FB-I (2005) survey and the SANHANES-1 (2012) survey, where 7.8% and 8.1% of children under 5 years were iron deficient. The current findings were even lower than Motadi et al. (2015), who reported that 7.2% and 28.1% of children were iron deficient. Iron deficiency during childhood is the form of nutritional deficiency that results from negative iron balance caused by insufficient dietary intake, absorption and utilisation of iron, increased requirements of iron during growth, blood loss because of infections from parasites such as malaria, soil-transmitted helminth infestations and schistosomiasis (Fançony et al. 2020).

### Limitation of the study

The current study did not determine whether the children had acute and chronic infections. Furthermore, inflammatory diseases, certain neoplastic diseases and liver disorders elevate serum ferritin because ferritin is an acute-phase protein (Gibson 2005; Gibson et al. 2010). The study focused only on Thulamela Municipality, and the findings may therefore not reflect general practices across Vhembe District and the province.

## Conclusion and recommendations

The study showed that the feeding practices and micronutrient status of the children were poor. This denotes that inappropriate or poor feeding practices can lead to poor micronutrient status, while appropriate feeding can improve micronutrient status and child survival.

Improving the nutritional status of children should follow an integrated approach tackling feeding practices, malnutrition and micronutrient deficiencies while considering the behavioural approach that will improve child survival and maternal health. Efforts should be made to improve infant feeding, emphasise locally available food (including indigenous food) and the importance of proper IYCF to reduce malnutrition among children. Nutrition professionals should plan and intensify public awareness of micronutrients and dietary diversity, especially in rural communities.

### Primary contribution of the study

This study highlights the feeding practices and micronutrient status of children less than 36 months. The results may contribute to the body of literature related to the feeding practices and micronutrients of children, and it has the potential to lead to improved appropriate feeding practices which, in turn, may improve the children’s micronutrient status and survival.
